# Equilibrium properties of *E. coli* lactose permease symport—A random-walk model approach

**DOI:** 10.1371/journal.pone.0263286

**Published:** 2022-02-04

**Authors:** Haoran Sun

**Affiliations:** School of Mathematical Sciences, Fudan University, Shanghai, China; Universite Cote d’Azur, FRANCE

## Abstract

The symport of lactose and H^+^ is an important physiological process in *E*. *coli*, for it is closely related to cellular energy supply. In this paper, we review, extend and analyse a newly proposed cotransport model that takes the “leakage” phenomenon (uncoupled particle translocation) into account and also satisfies the static head equilibrium condition. Then, we use the model to study the equilibrium properties, including equilibrium solution and the time required to reach equilibrium, of the symport process of *E*. *coli* LacY protein, when varying the parameters of the initial state of cotransport system. It can be found that in our extended model, H^+^ and lactose will reach their equilibrium state separately, and when “leakage” exists, it linearly affects the equilibrium solution, which is a useful property that the original model does not have. We later investigated the effect of the volume of periplasm and cytoplasm on the equilibrium properties. For a certain *E*. *coli* cell, as it continues to lose water and contract, the time for cytoplasm pH to be stabilized by symport increases monotonically when the cell survives. Finally, we reproduce the experimental data from a literature to verify the validity of the extension in this symport process. The above phenomena and other findings in this paper may help us to not only further validate or improve the model, but also deepen our understanding of the cotransport process of *E*. *coli* LacY protein.

## Introduction

LacY protein (lactose permease) of *Escherichia* *coli* is a kind of transport protein involved in the secondary active transport of hydrogen ions and lactose molecules. The protein uses the energy stored in the H^+^ electrochemical gradient to symport hydrogen ions into the cytoplasm along with β—galactosides, such as lactose [1]. Since the intracellular lactose concentration is usually higher than that in the environment, the above cotransport process is usually inverse to the lactose concentration gradient [2]. Possible mechanisms and mathematical models for the cotransport process have been studied for a long time, starting roughly from Cohen and Rickenberg’s report in 1955 [3]. Among the numerous research results so far, one of the most significant achievement is the “six-state” mechanism described by Kaback *et al*. in their 2001 article [4], which is based on a more universal cotransport mechanism proposed by Jardetzky in 1966 [5]. The so-called “six-state” mechanism refers to the fact that the cotransporter lactose permease have six different functional conformations (states) in the cotransport process. The LacY protein begins the reaction cycle at a outward-facing state 1, quickly binds a hydrogen ion, turning to state 2, then continues to trap a lactose molecule and turns to state 3, during which the cotransporter remains in the outward-facing state. After the combination of particles, the cotransporter makes a rapid conformational change to inward-facing, followed by detachment of the lactose molecule to state 5, then to state 6 by shedding the hydrogen ion, and finally returns to state 1 by another rapid conformational change. All above processes are naturally reversible. But further studies demonstrated that in fact there are actually 8 different conformations of lactose permease throughout the symport process, with another conformation 7 between states 3 and 4 and another conformation 8 between states 6 and 1 in the process described above [6]. Nevertheless, for modeling purposes only, these two new conformations can be merged into adjacent states with little effect on the results, so the six-state model is still often used to describe the symport of lactose permease.

However, the former conventional mechanism is challenged by some subsequent experimental results [7], where uncoupled transport is observed and determined, *i*.*e*., the two kinds of particles involved in cotransport do not follow the previous stoichiometric, and one kind has a uniport-like“leakage” phenomenon. In fact, similar incomplete H^+^/Sugar coupling phenomena have been widely observed for many members of the major facilitator superfamily, especially for the lactose permeases discussed in this paper [8], and there are several speculations and studies about the mechanisms. Even in the absence of the H^+^ electrochemical gradients on both sides of the membrane, the presence of symport of LacY protein can still be observed. For the symport of sodium ions with glucose by SGLT1 protein, which is similar to the cotransport process of *E*. *coli* LacY protein, several possible mechanisms leading to the leakage phenomenon have been proposed in the literature [9]. And as pointed out by molecular-dynamic simulations in the literature [10], the leakage phenomenon of the transporter vSGLT may be due to a kind of stochasticity in its intracellular release order of the two substrates, and a model was proposed for computational simulations using a similar idea in the literature [11]. It then poses a problem for how to modify previous existing mathematical models that simulate this “six-state” mechanism. Among these hypothetical mechanism the simpler one that has also been used many times to modify mathematical models, is to allow the transition between cotransporter states 2 and 5. Nevertheless, some literatures such as [12] propose that any determinate modified cotransport model by allowing the transition between states 2 and 5 cannot satisfy a certain thermodynamic condition (static head equilibrium condition). Then Barreto *et al*. propose a statistical mechanical model based on the above way of modifying conventional mechanism on the work of paper [13], which is a random-walk model and satisfies the static head equilibrium condition in the case of leakage [14].

Based on such a model, we are finally able to computationally simulate the transport process of *E*. *coli* LacY protein in the presence of leakage, and find some distinct equilibrium state properties in the presence and absence of leakage, such as the equilibrium solution and the convergence rate to the equilibrium solution. In this paper, we first briefly restate, extend and analyze the model in article [14] and predict the results of the computational simulations in the following. Next, we vary the intensity of the leakage, the volume of *E*. *coli* periplasm and cytoplasm and other parameters to study the variation of the corresponding equilibrium solution and the time required to reach equilibrium. Thus we find some very similar or different phenomena in the presence and absence of leakage. Finally, we illustrate the value of our changes to the original model with a realistic open system computational example, and discuss the problems encountered and possible improvements of this cotransport model in the computational simulation of the symport of LacY.

## Methods—Restatement, extension and analysis of the model

**Fig 1:**
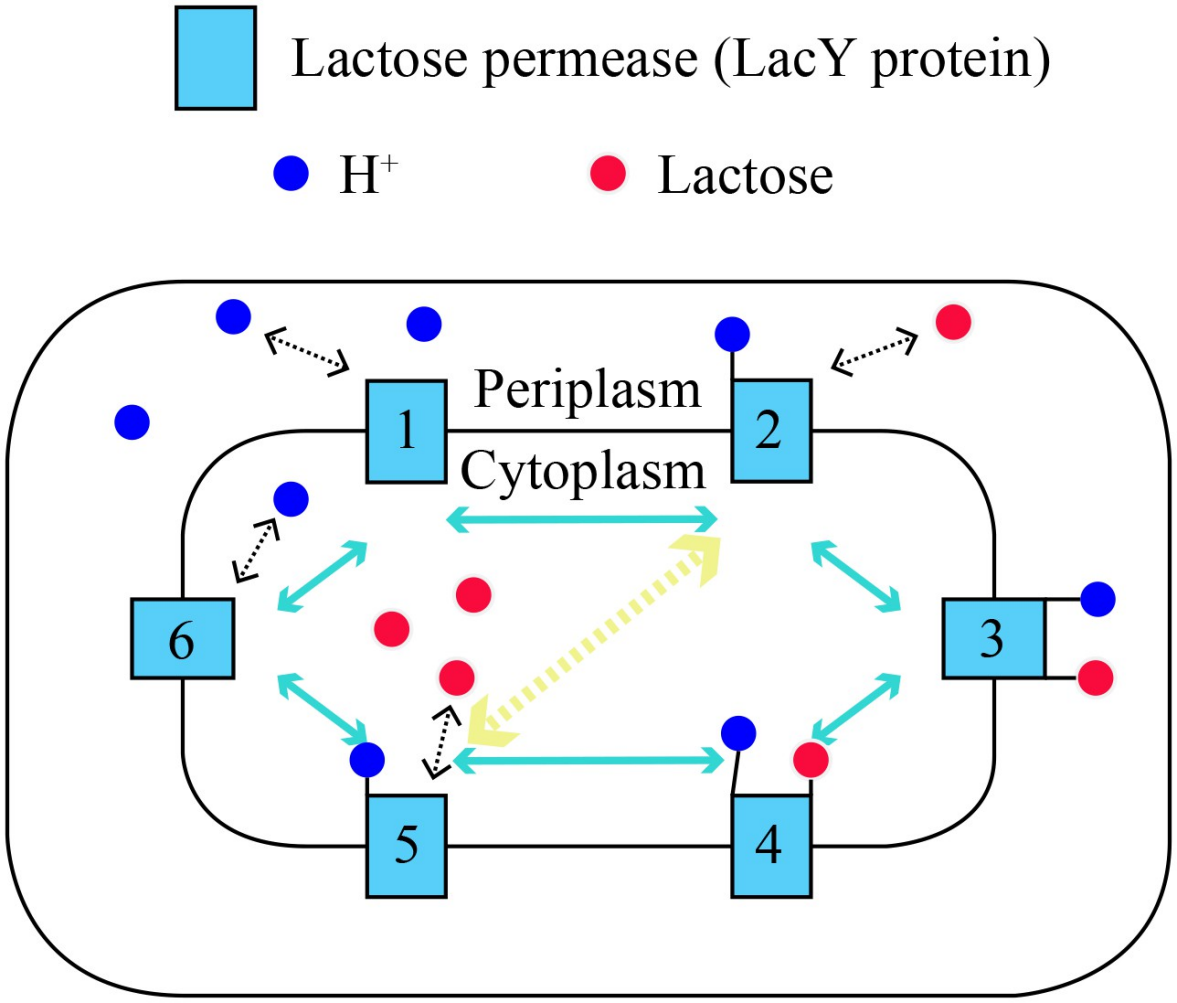
The modified six-state mechanism of cotransport. An alternating access model of *E*. *coli* lactose—H^+^ cotransport by Jardetzky modified with the transition between cotransporter states 2 and 5. A simple schematic of the “six-state” mechanism of cotransporter LacY protein of *E*. *coli* during cotransport is shown in the figure above. Numbers 1 to 6 indicate state 1 to state 6, which are the six effective conformations of LacY protein. The blue double-head arrows refer to the six states of cotransporter in relation to each other, and the yellow dashed arrow refers to the way to modify the present model—allowing the transition between cotransporter states 2 and 5.

The model in article [14] considers a closed system of periplasm and cytoplasm, the volumes of which are *V*_*p*_ and *V*_*c*_ respectively. Periplasm and cytoplasm are separated by a cell membrane with cotransporters embedded, across which there is a membrane potential ΔΨ that we assume a constant (cytoplasm lower). The total number of cotransporters on the cell membrane is fixed to *n*, and they are all assumed to be independent of each other. Let *n*_*k*_, *k* = 1, 2⋯6, represent the number of cotransporters in the current state *k*, respectively, and the sum of these six terms is clearly the fixed value *n*. Here I use an illustration Fig 1 to graphically show the six-state mechanism described in the introduction section for the readers to understand it more visually.

$N_{c}^{H^{+}}\left(t\right)$ and $N_{c}^{L}\left(t\right)$ represent the current number of hydrogen ions and lactose molecules in cytoplasm, and $N_{p}^{H^{+}}\left(t\right)$ and $N_{p}^{L}\left(t\right)$ similarly for periplasm. When it is not necessary to specify one kind of particle or one of periplasm and cytoplasm, we will later use *S* to refer to either of the two kinds of particles involved in cotransport and *l* refer to periplasm or cytoplasm. Since the system is closed, the total number of *S* particles in all states and positions is always equal to the number we put in at the beginning, as a constant value, denoted *N*_*S*_. *ξ* represents the intensity of leakage (similar to the ratio of leakage current to cotransport current excluding leakage) and takes the value in [0, 1]. *ξ* = 0 means no leakage occurs, *ξ* = 1 means the cotransporter in state 2 has an equal probability to take a conformation change to state 1,3 or 5, and the larger the value of *ξ*, the more obvious the leakage phenomenon is. The master equations of the model consists of ten differential equations related to each other, the exact form of which are shown as follows.

$$\begin{array}{c} \frac{dn_{k}}{dt}=\frac{1}{\tau_{0}}\sum_{k^{\prime }\neq k}\left(W_{k^{\prime }k}n_{k^{\prime }}-W_{kk^{\prime }}n_{k}\right),\;k=1,2,\cdots ,6\;, \end{array}$$

$$\begin{array}{c} \frac{1}{\nu_{S}}\frac{dN_{l}^{S}}{dt}=\frac{h}{\tau_{0}}\left(W_{K+1K}n_{K+1}-W_{KK+1}n_{K}\right). \end{array}$$

*ν*_*S*_ is the number of particle *S* transported in one cycle of cotransporter in Fig 1, here equals 1 for H^+^ and lactose. The values of *h* and *K* depend on *l* and *S*, in particular, *h* = 1 and *K* = 2 − *f* for $N_{p}^{A}$; *h* = 2*f* − 1 and *K* = 1 + *f* for $N_{p}^{B}$; *h* = −1 and *K* = 4 + *f* for $N_{c}^{A}$; *h* = 1 − 2*f* and *K* = 5 − *f* for $N_{c}^{B}$. *τ*_0_ is the average time for a cotransporter to change its state. The transition probabilities *W*_*ij*_ for *i*, *j* = 1, 2, ⋯, 6 can be combined into the following transition matrix,

$$(\begin{array}{cccccc} W_{11} & \gamma_{p}\alpha_{12} & 0 & 0 & 0 & \frac{1}{2}\alpha_{16}\\ \delta_{p}\alpha_{21} & W_{22} & \epsilon_{p}\alpha_{23} & 0 & \frac{\xi }{3}\alpha_{25} & 0\\ 0 & \frac{1}{2}\alpha_{32} & W_{33} & \frac{1}{2}\alpha_{34} & 0 & 0\\ 0 & 0 & \frac{1}{2}\alpha_{43} & W_{44} & \frac{1}{2}\alpha_{45} & 0\\ 0 & \frac{\xi }{3}\alpha_{52} & 0 & \epsilon_{c}\alpha_{54} & W_{55} & \delta_{c}\alpha_{56}\\ \frac{1}{2}\alpha_{61} & 0 & 0 & 0 & \gamma_{c}\alpha_{65} & W_{66}\; \end{array}).$$

*α*_*pq*_ are all constants decided by *S* and *l*, and the details are shown in article [14]. *W*_*kk*_ can be achieved by the relation $\sum_{k^{\prime }=1}^{6}W_{kk^{\prime }}=1$ and the parameters *γ*_*l*_, *δ*_*l*_, and *ϵ*_*l*_ take the forms

$$\begin{array}{c} \gamma_{l}=\frac{1}{2}\left(1-f+f\pi_{l}^{A}\right),\delta_{l}=\frac{1}{2}(1-\frac{\xi }{3})\left[f+\left(1-f\right)\pi_{l}^{B}\right],\epsilon_{l}=\frac{1}{2}(1-\frac{\xi }{3})\left[f\pi_{l}^{B}+\left(1-f\right)\pi_{l}^{A}\right], \end{array}$$

where *f* = 0 *or* 1 for antiport or symport, and the exact forms of $\pi_{l}^{S}$ can be found in the original [14].

Here we will first make a small correction to the analysis in [14]. The original [14] first makes the left-hand side quotient of the master equation tend to 0 by making *t* → ∞, which causes all variables to take equilibrium solutions (or asymptotic solutions), and the solutions when *ξ* = 0 and *ξ* ≠ 0 have the following form,

$$\begin{array}{c} N_{l,\xi =0}^{S}=C_{0}V_{l}{\left(\frac{\alpha_{K+1K}}{\alpha_{KK+1}}\frac{n_{K+1}}{n_{K}}\right)}^{h/\nu_{S}}, \end{array}$$

$$\begin{array}{c} N_{l,\xi \neq 0}^{S}={\left(1-\frac{\xi }{3}\right)}^{\tilde{h}/\nu_{S}}C_{0}V_{l}{\left(\frac{\alpha_{K+1K}}{\alpha_{KK+1}}\frac{n_{K+1}}{n_{K}}\right)}^{h/\nu_{S}}. \end{array}$$

$\tilde{h}$ is also a constant for a certain kind of particle *S*. After obtaining the above results, the paper [14] states that $N_{l,\xi \neq 0}^{S}={\left(1-\frac{\xi }{3}\right)}^{\tilde{h}/\nu_{S}}N_{l,\xi =0}^{S}$, which leads to the equilibrium solution for all values of *ξ*. However, a careful observation shows that the above two equations are essentially the relationship between the equilibrium solution of the particle numbers $N_{l,\xi }^{S}$ and the cotransporter numbers *n*_*K*_. The equilibrium solutions of the cotransporters are not necessarily the same for different values of *ξ* (in practice, it is clear from the calculations below that they are indeed not the same), so the conclusion of the original equilibrium solution in [14] does not hold.

In fact, we can get new information about the equilibrium solution by the Gibbs free energy change as well as the electrochemical gradient. The original [14] proves that the equilibrium solution satisfies the following static head equilibrium condition when *ξ* = 0,

$$\begin{array}{c} \frac{N_{c}^{A}/V_{c}}{N_{p}^{A}/V_{p}}={\left(\frac{N_{p}^{B}/V_{p}}{N_{c}^{B}/V_{c}}\right)}^{\left(2f-1\right)\nu_{B}/\nu_{A}}\times \text{exp}(-\frac{1}{k_{B}T}[Q_{A}+\left(2f-1\right)\frac{\nu_{A}}{\nu_{B}}Q_{B}]\Delta \Psi ). \end{array}$$

Here *f* = 1 for lactose permease and *A*, *B* are particles transported by cotransporter. And the following scaling relationships between equilibrium solutions are obtained in the same way when *ξ* ≠ 0 (also satisfying the static head equilibrium),

$$\begin{array}{c} \frac{N_{c}^{S}/V_{c}}{N_{p}^{S}/V_{p}}=\text{exp}(-\frac{1}{k_{B}T}Q_{S}\Delta \Psi ). \end{array}$$

*Q*_*S*_ is the charge of a particle *S*, *k*_*B*_ is the Boltzmann constant, and *T* is the is the ambient temperature of *E*. *coli*. The above two relations are important constraints on the equilibrium solution, since in reality the number of cotransporters is much smaller than the number of particles involved in cotransport, and the total number of particles is constant during the transport, *i*.*e*. the following two equations hold at all times during the transport,

$$\begin{array}{c} N_{A}-N_{c}^{A}-N_{p}^{A}=\nu_{A}\left[n_{3}+n_{4}+\left(n_{2}+n_{5}\right)f\right], \end{array}$$

$$\begin{array}{c} N_{B}-N_{c}^{B}-N_{p}^{B}=\nu_{B}\left[\left(n_{3}+n_{4}\right)f+\left(1-f\right)\left(n_{1}+n_{6}\right)\right]. \end{array}$$

Then we can see that under the general condition that the total number of cotransporters is very small, the equilibrium solution changes quite little with the parameter *ξ* when *ξ* ≠ 0. Only if the total number of cotransporters is not small relative to the total number of particles and also the distribution of cotransporters in each state varies considerably when *ξ* changes does the equilibrium solution change significantly. The computational verification of the above theoretical descriptions will be carried out below.

The above conclusions give directional hints for our changes to the model. As stated in [14], the factors related to *ξ* in all *W*_*ij*_ originate from the assumption that at each time step Δ*t*, a cotransporter in state *k* can change to *k* + 1 or *k* − 1 with equal probability amplitudes. And for *k* = 1, 3, 4, and 6, cotransporter can only move to state *k* + 1 or *k* − 1, while state 2 and 5 can move to *k* + 1, *k* − 1 and each other. In [14], the authors assume that state 2 to 5 and state 5 to 2 share the same propability amplitude, thus the first component of *W*_25_ and *W*_52_ is *ξ*/3. So we can extend the model, that is, let the first component of *W*_25_ be *ξ*/3 and the first component of *W*_52_ be *η*/3 that need not be equal to *ξ*. The specific changes in the transition matrix are as follows,

$$\begin{array}{c} W_{52}:\;\frac{\xi }{3}\alpha_{52}\to \frac{\eta }{3}\alpha_{52},\;W_{54}:\;\epsilon_{c}\alpha_{54}\to \phi_{c}\alpha_{54},\;W_{56}:\;\delta_{c}\alpha_{56}\to \psi_{c}\alpha_{56}, \end{array}$$

in which $\psi_{c}=\frac{1}{2}(1-\frac{\eta }{3})\left[f+\left(1-f\right)\pi_{c}^{B}\right],\;\phi_{c}=\frac{1}{2}(1-\frac{\eta }{3})\left[f\pi_{c}^{B}+\left(1-f\right)\pi_{c}^{A}\right]$, and *η* ∈ [0, 1]. In fact, the above changes are not made arbitrarily, the extended model still satisfies the static head equilibrium condition, which is a very important contribution of the original model. The proof of this assertion is similar to the original that we restated before. The equilibrium solution of our extended model are as follows,

$$\begin{array}{c} N_{p,\xi \neq 0,\eta \neq 0}^{S}={\left(1-\frac{\xi }{3}\right)}^{\tilde{h}/\nu_{S}}C_{0}V_{p}{\left(\frac{\alpha_{K+1K}}{\alpha_{KK+1}}\frac{n_{K+1}}{n_{K}}\right)}^{h/\nu_{S}}, \end{array}$$

$$\begin{array}{c} N_{c,\xi \neq 0,\eta \neq 0}^{S}={\left(1-\frac{\eta }{3}\right)}^{\tilde{h}/\nu_{S}}C_{0}V_{c}{\left(\frac{\alpha_{K+1K}}{\alpha_{KK+1}}\frac{n_{K+1}}{n_{K}}\right)}^{h/\nu_{S}}. \end{array}$$

Then using the chemical potential relationship presented in the original, our assertion can be verified.

Now we also need to show that such a change is valuable, i.e., that *ξ* and *η* have different effects on the computational results of the model, especially the equilibrium solution and the time needed to reach equilibrium. First, it should be pointed out that the model has a unique equilibrium solution for any set of reasonable initial values if *ξ* and *η* are fixed constants in [0, 1], and that equilibrium solution is almost continuously dependent on the parameters *ξ* and *η*. In other words, for a fixed set of initial conditions, when *ξ* → *ξ*_0_ ≠ 0, *η* → *η*_0_ ≠ 0, the equilibrium solution tends to that when *ξ* = *ξ*_0_, *η* = *η*_0_. And also, in general we prefer to study initial values that are predicted to produce significant transition phenomena, that is, cases where the equilibrium solution differs significantly from the initial value. As can be seen as an example in our later calculations for the *E*. *coli* lactose permease symport of H^+^ and lactose molecules, such an initial value leads to a large difference between the equilibrium solution when *ξ* = *η* = 0 and that when *ξ* ≠ 0, *η* ≠ 0. Since *ξ* and *η* are in opposite positions and controlling the two directions of the mutual transition of cotransporter states 2 and 5 respectively, the equilibrium solutions from either the cases when *ξ* = 0, *η* ≠ 0 or those when *ξ* ≠ 0, *η* = 0 must be close to the cases when *ξ* = *η* = 0, while the other close to the cases when *ξ* ≠ 0, *η* ≠ 0. At this point we can say that the two parameters *ξ* and *η* do have different effects on the equilibrium properties of the extended model, one determining the overall trend and the other allowing for fine-tuning of the results on this basis. In the following we will use the symporting process of H^+^ and lactose by *E*. *coli* lactose permease as an example to verify these claims we made above.

Then a new question arises, how could we estimate the values of the two parameters *ξ* and *η* for a specific cotransport process, for the methods given at the end of the original [14] may not work in the extended model. We will give a brief discussion about this at the end of the article.

Finally, we need to clarify that the above model only considers the effect of cotransport processes on particle concentration and does not consider the effect of other intracellular life activities, such as the breakdown of lactose in cytoplasm by β—galactosides and many mechanisms that maintain pH homeostasis in the cell. If these effects on particle concentrations are integrated into the model, since there are fixed chemical equilibrium relationships for all these reactions, their effects would be almost equivalent to multiplying a fixed constant on the particle concentrations in cytoplasm. However, the new parameters introduced in this way pose the problem of poor estimation, and also these constants do not have much practical impact on the problem studied in this paper from a mathematical point of view even if they are not considered. Furthermore, there are few similar studies that include these above factors in their models for calculation, perhaps also for reasons of their over complexity and little relevance. For all these reasons, the model in this paper does not consider processes other than cotransport that affect particle concentrations, but it may indeed make the model more accurate if these factors are taken into account, and this is also a direction for further improvement of the model.

## Results—Computational simulations and equilibrium property studies using the above model

In the following of this paper, computational simulations of the *E*. *coli* LacY protein 1:1 symport processes of H^+^ and lactose are performed with the aforementioned model. The values used in the calculation are from the article [13, 14] or selected according to the reality, as detailed in the calculation section later. We use the finite difference method to solve the differential master equations of the model numerically [15], that is, we use the relation *dN*(*t*)/*dt* ≈ (*N*(*t* + Δ*t*) − *N*(*t*))/Δ*t* to transform the differential equation into a difference equation for calculation, and in subsequent calculations in this paper we take the time step Δ*t* = 0.1*τ*_0_. We will first give the results of all simulations in this section, and then discuss and analyze them all together in the next section.

### Effect of parameters *ξ* and *η* (leakage intensity) on equilibrium properties

**Fig 2:**
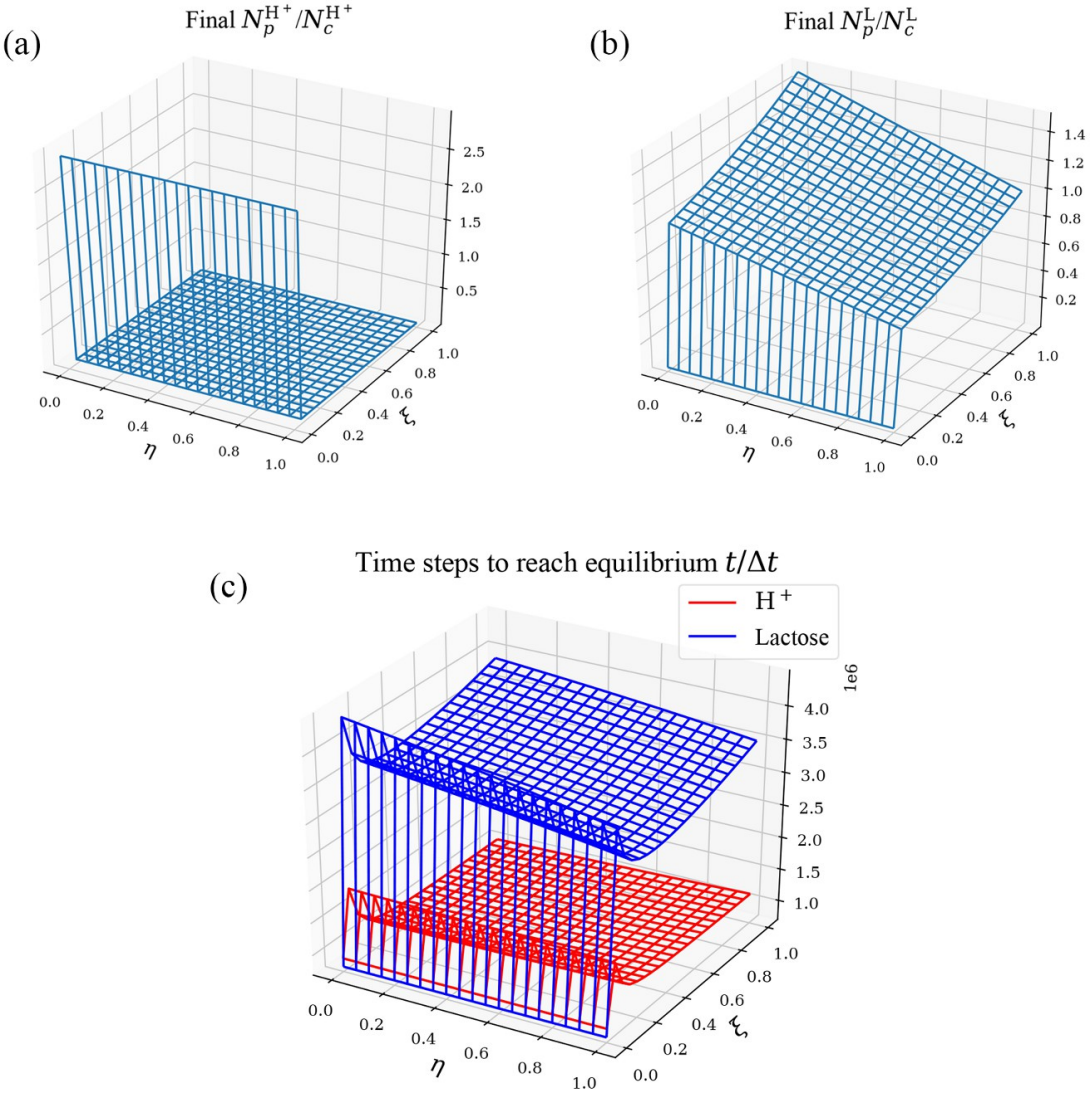
Equilibrium properties of lactose permease symport in *E*. *coli* as *ξ* and *η* changes. The cases are shown as *ξ* and *η* takes the twenty partition points of interval [0, 1](0.05 × *i*, *i* = 0, 1, ⋯, 20) with the following initial conditions: $n_{1}=10^{3},n_{k\neq 1}=0,N_{p}^{H^{+}}=10^{5},N_{p}^{L}=2.5\times 10^{4},N_{c}^{L}=7.5\times 10^{4},N_{c}^{H^{+}}=10^{3}$. And also, we set *V*_*c*_ = *V*_*p*_ = 10^6^/*C*_0_, *C*_0_ is a constant with the unit of nm^−3^. The meaning of *C*_0_ is the same as which in [14], similarly hereinafter. (a) The value of $N_{p}^{H^{+}}$/$N_{c}^{H^{+}}$ at equilibrium. (b) The value of $N_{p}^{L}$/$N_{c}^{L}$ at equilibrium. (c) Time used to reach equilbrium state for H^+^ and lactose with the unit of Δ*t*.

As we just mentioned above, parameters *ξ* and *η* have different positions in the model and do not play exactly the same role. The following Fig 2 shows the effects of *ξ* and *η* on the equilibrium solution of H^+^ and lactose, and the time used for cotransporting from the initial state to the equilibrium state, when the initial conditions are fixed.

The reason why the initial conditions of H^+^ being so is that, according to the article [16], *E*. *coli* cells are generally in an environment that causes the pH of periplasm about 1.8 to 2.1 lower than that of cytoplasm. As for the initial value of lactose, we design it to be higher than the actual condition while maintaining the ratio of intracellular to extracellular concentrations, which will make some specific phenomena we are interested in manifest more clearly. Such a design does not change the essence of the simulation significantly.

Although the equilibrium solution is asymptotic and theoretically not achievable in finite time, we can still consider that equilibrium has been reached when the change rate or the first-order backward difference quotient of the solution is small enough in our simulation. When there is no leakage (*ξ* = *η* = 0), it is easy to find that H^+^ and lactose reach equilibrium at the same time in error permissible by calculation, which is also easy to see from the above theoretical analysis. However, when leakage does exist (*ξ* ≠ 0, *η* ≠ 0), the time for the two kinds of particles to reach equilibrium is seperated. The equilibrium time of the hydrogen ion changes rapidly from monotonically decreasing with *η* to monotonically increasing as *ξ* increases, while monotonically decreasing with *ξ* always holds. For lactose, the equilibrium time remains monotonically decreasing as *ξ* or *η* increasing. As it would become very mathematical and tedious to try to explain such phenomena in their entirety, so instead of explaining it as a whole, we will just do the following more detailed analysis of the special case of *ξ* = *η*, which is the original model.

**Fig 3:**
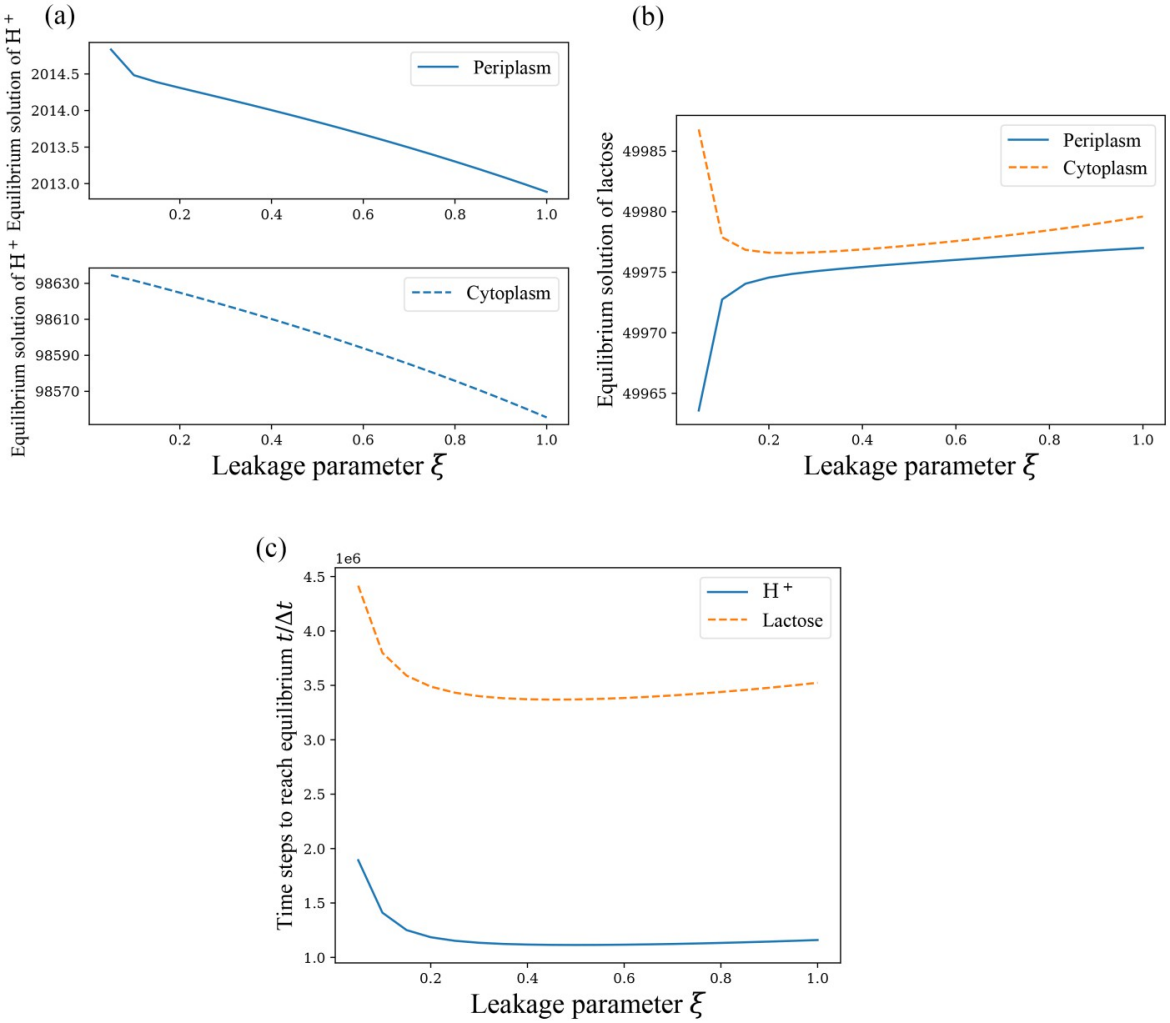
Equilibrium properties of lactose permease symport in *E*. *coli* when *ξ* = *η*. Only the *ξ* ≠ 0 cases are shown with the initial conditions the same as Fig 2. The vertical coordinate represents the number of particles in (a) and (b). (a) The equilibium solution of H^+^ in periplasm and cytoplasm. (b) The equilibium solution of lactose in periplasm and cytoplasm. (c) Time used to reach equilbrium state for H^+^ and lactose with the unit of Δ*t*.

The *ξ* = *η* = 0 and *ξ* = *η* ≠ 0 cases are too different to be studied together, and the *ξ* = 0 cases are shown before, so we focus on the *ξ* = *η* ≠ 0 case. First, the correlation between the equilibrium solution and *ξ* will be verified. The following Fig 3 shows the correlation between *ξ* and the equilibrium solutions as well as that between *ξ* and the time to reach equilibrium states for H^+^ and lactose at a fixed initial condition, respectively.

The above Fig 3(c) shows the relationship between the time of two kinds of particles to reach equilibrium and *ξ* when *ξ* ∈ (0, 1]. Fig 3(c) is calculated by considering that when $\left[N_{c}^{S}\left(t\right)-N_{c}^{S}\left(t-\Delta t\right)\right]/N_{c}^{S}\left(t\right)\leq 10^{-9}$ holds for almost every discrete time moments from some t (for over 99.9999% of time moments), the particle *S* reach the equilibrium. Subsequent calculations of the time to reach the equilibrium are similar, but accuracy may be adjusted as needed. The reasons for not using the first-order backward difference quotient and instead using change rate are the poor estimation of the range of the difference quotient and the fact that the difference quotient in this model is not guaranteed to be consistently smaller than the required accuracy after a certain time.

Since we have studied the effect of parameter *η* and the difference between *ξ* and *η* above, we can just consider the special case of *ξ* = *η* when we study the effect of other factors below.

### Effect of periplasm and cytoplasm volumes *V*_*p*_, *V*_*c*_ on equilibrium properties

**Fig 4:**
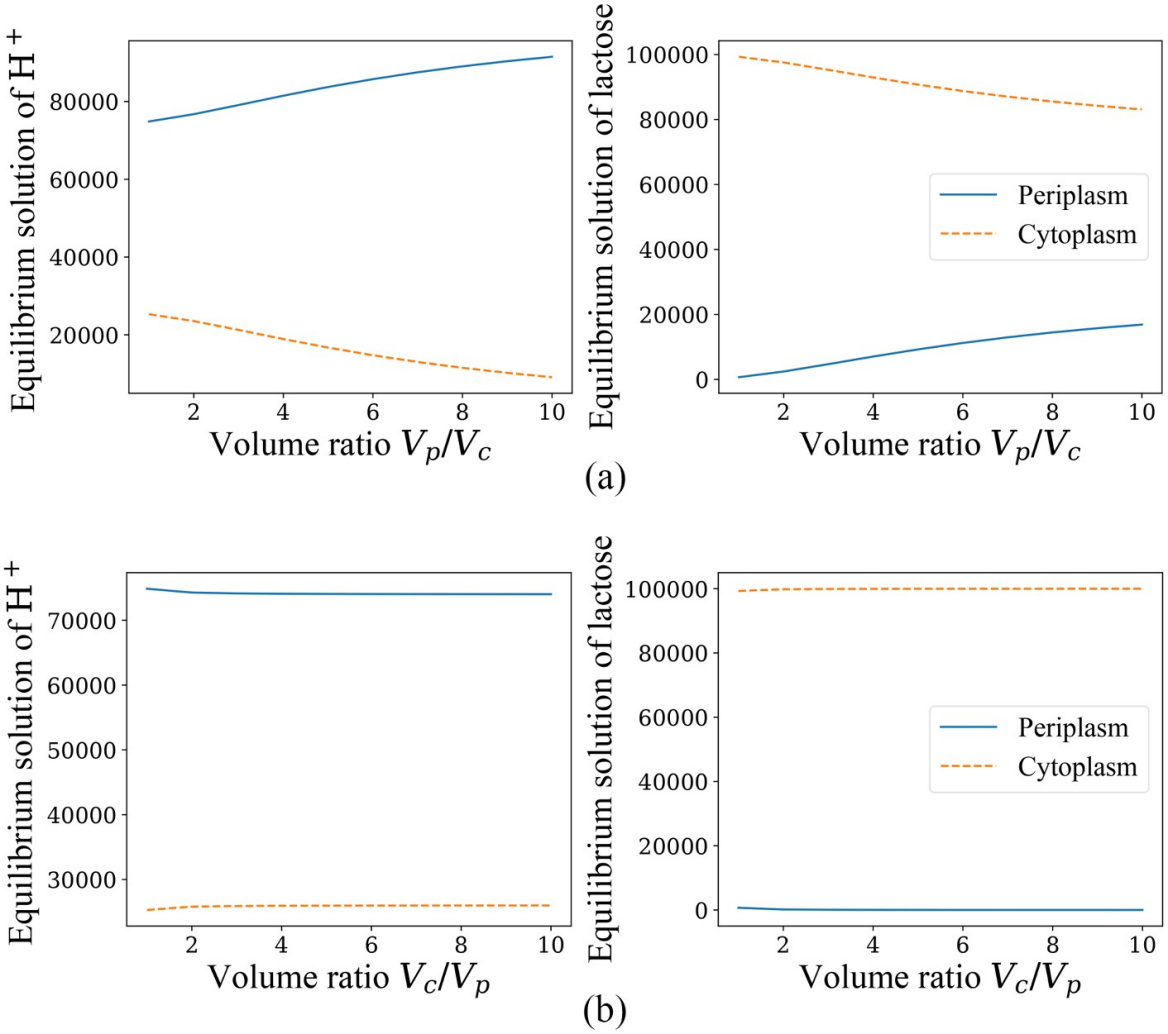
The equilibium solutions for *ξ* = 0 when *V*_*c*_ or *V*_*p*_ is fixed and the other one varies. (a) The equilibium solutions of H^+^ and lactose when *V*_*c*_ is fixed to 10^6^/*C*_0_ and *V*_*p*_/*V*_*c*_ > 1, with initial conditions $n_{1}=10^{3},n_{k\neq 1}=0,N_{p}^{H^{+}}=10^{5}$, $N_{p}^{L}=2.5\times 10^{4}$, $N_{c}^{L}=7.5\times 10^{4},N_{c}^{H^{+}}=10^{3}$. (b) The equilibium solutions of H^+^ and lactose when *V*_*c*_ is fixed to 10^6^/*C*_0_ and *V*_*p*_/*V*_*c*_ < 1, with the same initial conditions before.

The previous arithmetic examples have been calculated assuming *V*_*p*_ = *V*_*c*_, and as seen before, the proportionality satisfied by the equilibrium solution when *ξ* ≠ 0 is actually a proportionality between the particle concentrations in the two reaction chambers (periplasm and cytoplasm), so we consider changing the periplasm and cytoplasm volume ratios to verify the conclusion and observe if there are new phenomena. Also due to the previous conclusions on the equilibrium solution and the convergence rate in the case of *ξ* ≠ 0, we can only study the *ξ* = 0 and *ξ* = 1 cases. Let’s first examine the equilibrium solution from a mathematical point of view, and the equilibrium solutions of the *ξ* = 0 case are shown in the following Fig 4.

From Fig 4, the following phenomenon can be found that under the condition of fixing the number of particles involved in cotransport (*i*.*e*. fixing $N_{c}^{S},N_{p}^{S}$ in initial conditions), the change of *V*_*c*_ seems to have less effect on the equilibrium solution than *V*_*p*_, and the system equilibrium solution is more insensitive to the change of *V*_*c*_. When changing other initial conditions and making *V*_*p*_ a constant value to change *V*_*c*_/*V*_*p*_, this phnomenon is actually more accurately formulated as that changing *V*_*c*_ or *V*_*p*_ only under the condition that *V*_*p*_/*V*_*c*_ > 1 has a more pronounced effect on the equilibrium solution when *ξ* = 0, thus showing an insensitivity to the increase in the denominator *V*_*c*_. After adjusting the parameters and performing a lot of calculations, it is found that this property is somewhat related to the membrane potential. In this case the direction of potential decrease is from periplasm to cytoplasm, and the membrane potential is −100*mV*. However, when decreasing this potential difference, this phenomenon becomes less and less obvious, especially when reversing the membrane potential, *i*.*e*. making the potential of cytoplasm higher than that of periplasm, the significances of *V*_*c*_ and *V*_*p*_ in the above properties are switched, and *V*_*p*_ becomes the one that has less influence on the equilibrium solution of the system.

**Fig 5:**
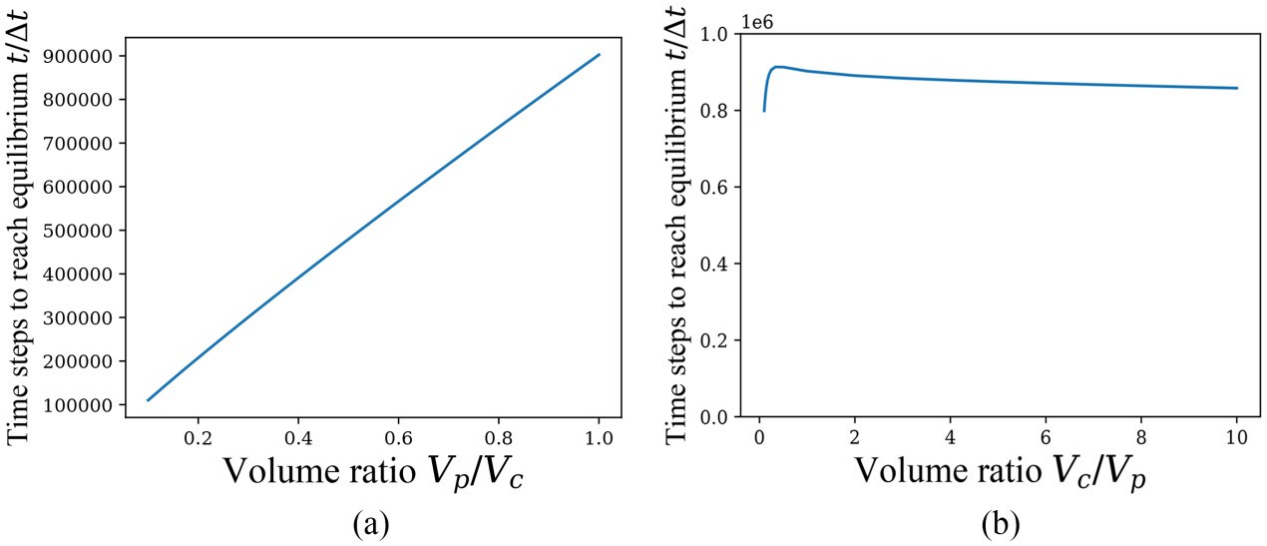
Time to reach equilibrium for *ξ* = 0 when *V*_*c*_ or *V*_*p*_ is fixed and the other one varies. Time used to reach equilbrium state with the unit of Δ*t* when the initial concentrations of H^+^ and lactose are fixed in periplasm and cytoplasm, which has the initial conditions $n_{1}=10^{3},n_{k\neq 1}=0,N_{p}^{H^{+}}/V_{p}=C_{0}/10,N_{c}^{H^{+}}/V_{c}=C_{0}/1000,N_{p}^{L}/V_{p}=C_{0}/40,N_{c}^{L}/V_{c}=3C_{0}/40$, *ξ* = 0. (a) *V*_*c*_ fixed to 10^6^/*C*_0_. (b) *V*_*p*_ fixed to 10^6^/*C*_0_.

Next, according to common biological sense, we will observe the relationship between *V*_*p*_/*V*_*c*_ or *V*_*c*_/*V*_*p*_ and the time required for the system to reach equilibrium when *V*_*p*_/*V*_*c*_ < 1. In the following, the case of *ξ* = 0 comes first. Inspired by the above phenomena, we fix *V*_*c*_ and *V*_*p*_ separately and change the other one to calculate. For practical reasons, particle numbers should not be fixed as in the case of equilibrium solution just probed before, but the concentration of particles in periplasm and cytoplasm in initial conditions should be fixed, so that the results are more realistic. The following Fig 5(a) and 5(b) are plotted with *V*_*p*_/*V*_*c*_ as the horizontal coordinate (with *V*_*c*_ fixed) and *V*_*c*_/*V*_*p*_ as the horizontal coordinate (with *V*_*p*_ fixed), respectively, and the time to reach the equilibrium solution as the vertical coordinate.

When the change rate of $N_{c}^{S}$ is always less than 10^−9^, the particle *S* is considered to reach equilibrium in the above Fig 5. It is still clear to find different effects of *V*_*p*_ and *V*_*c*_ on the time required to reach equilibrium, and the effect of *V*_*c*_ is still much smaller than that of *V*_*p*_. Increasing *V*_*p*_ when *V*_*c*_ is fixed can monotonically increase the time for the system to reach equilibrium. However, changing *V*_*c*_ when *V*_*p*_ is fixed has a less significant effect on the convergence rate.

**Fig 6:**
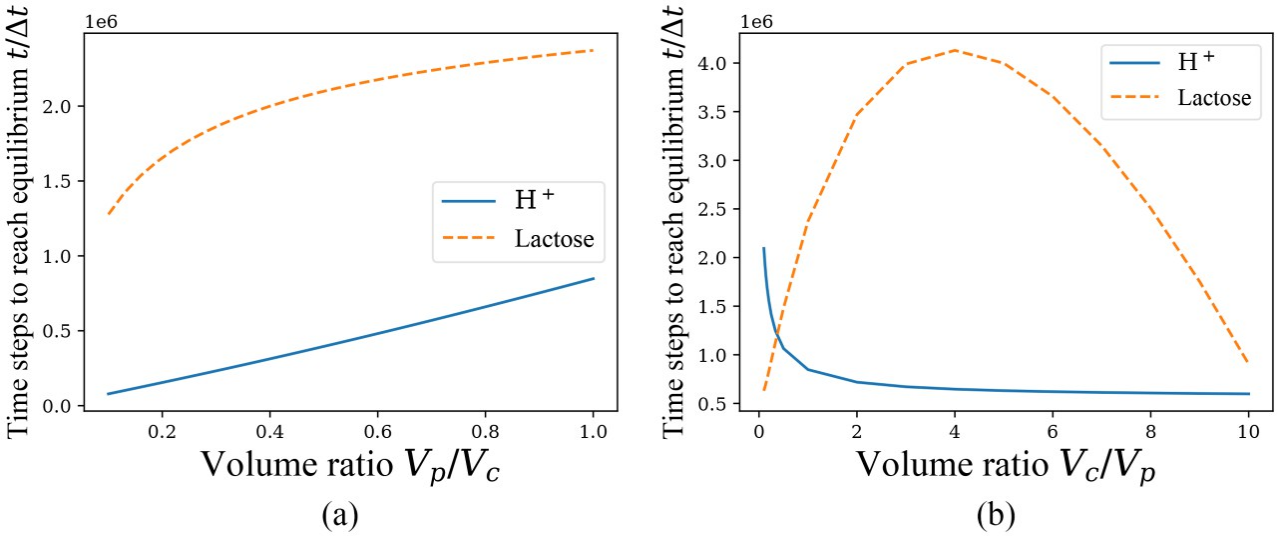
Time to reach equilibrium for *ξ* = 1 when *V*_*c*_ or *V*_*p*_ is fixed and the other one varies. Different time for H^+^ and lactose to reach equilbrium state with the unit of Δ*t* when the initial concentrations of H^+^ and lactose are fixed in periplasm and cytoplasm, which has the initial conditions $n_{1}=10^{3},n_{k\neq 1}=0,N_{p}^{H^{+}}/V_{p}=C_{0}/10,N_{c}^{H^{+}}/V_{c}=C_{0}/1000,N_{p}^{L}/V_{p}=C_{0}/40,N_{c}^{L}/V_{c}=3C_{0}/40$, *ξ* = 1. (a) *V*_*c*_ fixed to 10^6^/*C*_0_. (b) *V*_*p*_ fixed to 10^6^/*C*_0_.

The above is the case of *ξ* = 0. And for the case of *ξ* = 1, it is verified that the conclusion about the ratio of equilibrium solutions, *i*.*e*., $\left(N_{c}^{S}/V_{c}\right)/\left(N_{p}^{S}/V_{p}\right)=\text{const}.$, holds for both H^+^ and lactose particles within error permissibility when fixing the number of each particle in the initial condition. Then the next concern is the time required to reach the equilibrium solution when *ξ* = 1, and we still require a fixed concentration of the particles in periplasm and cyptoplasm in the initial conditions. The following figures Fig 6(a) and 6(b) show the relations between *V*_*p*_/*V*_*c*_ (*V*_*c*_ fixed) or *V*_*c*_/*V*_*p*_ (*V*_*p*_ fixed), and the time to reach the equilibrium solution, respectively, and we consider that the particle *S* reaches equilibrium when the rate of change of $N_{c}^{S}$ is always less than 10^−8^.

It is easy to see that in the case of *ξ* = 1 there are some phenomena that do not match our expectations. For example, with *V*_*c*_ fixed, the time to reach equilibrium for both kinds of particles increase monotonically with the increase of *V*_*p*_; however, with *V*_*p*_ fixed, the equilibrium time for H^+^ decreases monotonically with *V*_*c*_ increasing, but the equilibrium time for lactose shows an increase followed by a decrease and takes a maximum around *V*_*c*_/*V*_*p*_ = 4.

**Fig 7:**
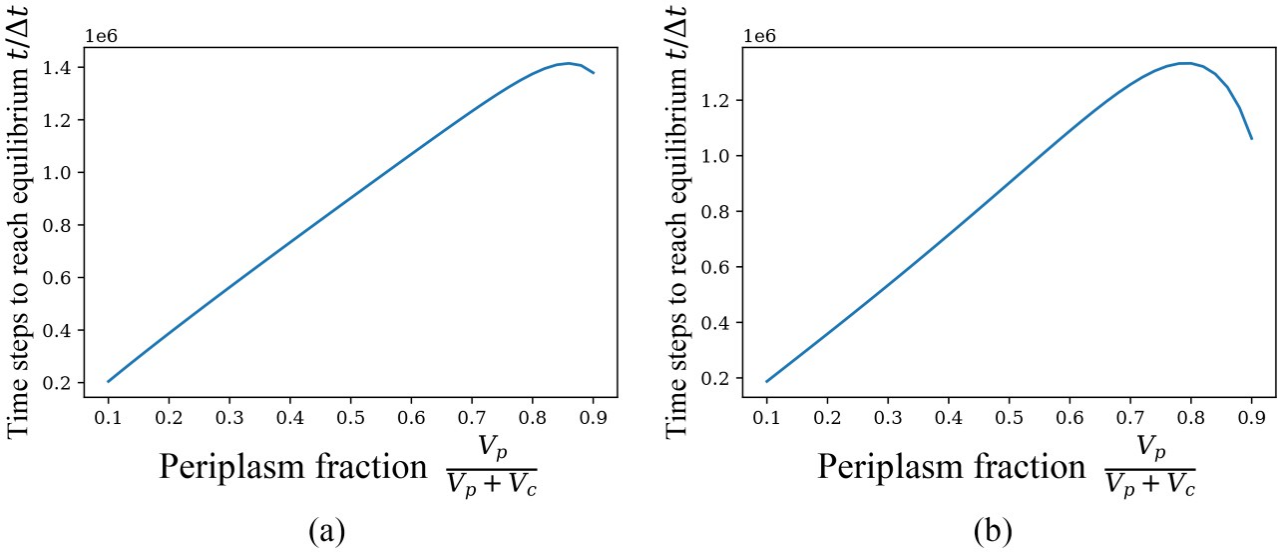
Time to reach equilibrium with the variation of periplasm fraction $\frac{V_{p}}{V_{p}+V_{c}}$, *ξ* = 0. The sum of volumes of periplasm and cytoplasm is fixed, *V*_*c*_+ *V*_*p*_ = 2 × 10^6^/*C*_0_. (a) Initial particle concentration fixed, and the initial conditions are like $n_{1}=10^{3},n_{k\neq 1}=0,N_{p}^{H^{+}}/V_{p}=C_{0}/10,N_{c}^{H^{+}}/V_{c}=C_{0}/1000,N_{p}^{L}/V_{p}=C_{0}/40,N_{c}^{L}/V_{c}=3C_{0}/40$. (b) Initial particle population fixed, and the initial conditions are like $n_{1}=10^{3},n_{k\neq 1}=0,N_{p}^{H^{+}}=10^{5},N_{p}^{L}=2.5\times 10^{4},N_{c}^{L}=7.5\times 10^{4},N_{c}^{H^{+}}=10^{3}$.

Above we set the volume ratio of periplasm and cytoplasm as the horizontal coordinate to calculate, but for a fixed *E*. *coli* cell (or cells from the same population), periplasm is the portion between the cell wall (cytoderm) and the cell membrane, and cyptoplasm is the portion within the cell membrane other than the nuclear region, and the sum of the volumes *V*_*c*_ + *V*_*p*_ varies very little due to the rigidity of the cytoderm. Therefore, we continue to investigate its effect on the convergence speed by taking the percentage of periplasm to the sum of periplasm and cyptoplasm volumes as the horizontal coordinate while fixing the sum of the two volumes. In this case, we choose to fix the initial concentration and initial number of particles in periplasm and cyptoplasm, respectively, and calculate the time required for different particles to reach equilibrium. The following Fig 7 is the image of the time required to reach equilibrium as the periplasm fraction changes for the case *ξ* = 0, where it is still considered that the particle *S* reaches the equilibrium solution when the change rate of $N_{c}^{S}$ is always less than 10^−9^. As seen from the experimental data in article [17, 18], the range of horizontal ordinates in Fig 7 already includes the volume fraction of periplasm (8%-40%) in which *E*. *coli* can survive.

It can be found that the two graphs have some similarity, both appearing to be approximately linearly increasing followed by monotonically decreasing, but within the realistic range (8%-40%), the time required for equilibrium increases monotonically with the increase in the volume fraction of periplasm. Although it may seem strange, the above case of *ξ* = 0 can actually be roughly explained by the standard theoretical methods, as shown below in the discussion section.

**Fig 8:**
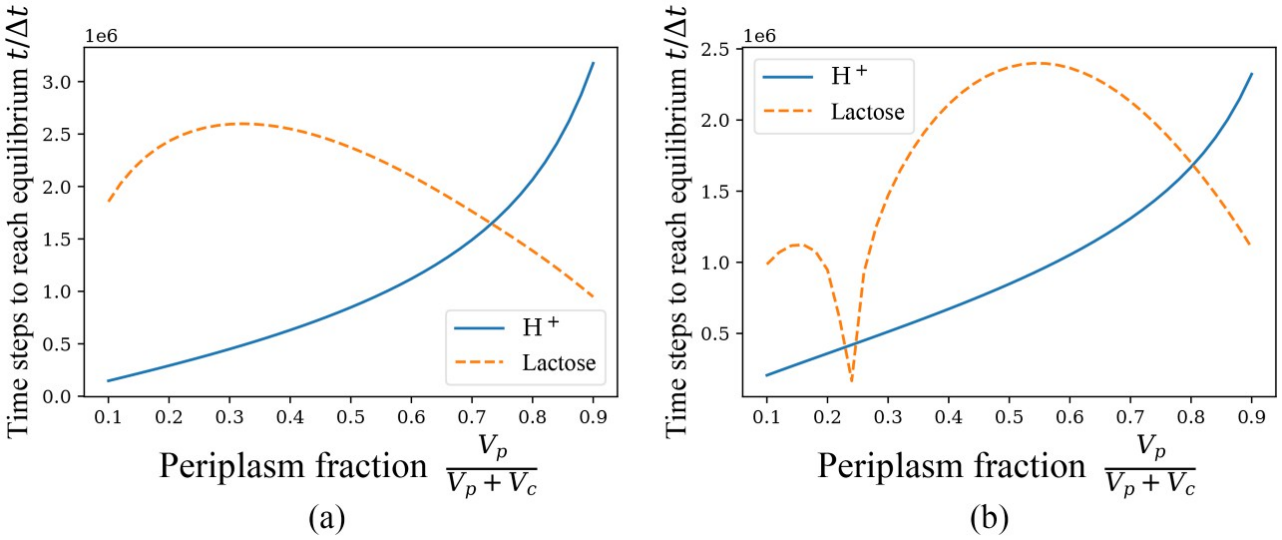
Time to reach equilibrium with the variation of periplasm fraction $\frac{V_{p}}{V_{p}+V_{c}}$, *ξ* = 1. Different time for H^+^ and lactose to reach equilbrium state with the unit of Δ*t*. The sum of volumes of periplasm and cytoplasm is fixed, *V*_*c*_+ *V*_*p*_ = 2 × 10^6^/*C*_0_. (a) Initial particle concentration fixed, and the initial conditions are like $n_{1}=10^{3},n_{k\neq 1}=0,N_{p}^{H^{+}}/V_{p}=C_{0}/10,N_{c}^{H^{+}}/V_{c}=C_{0}/1000,N_{p}^{L}/V_{p}=C_{0}/40,N_{c}^{L}/V_{c}=3C_{0}/40$. (b) Initial particle population fixed, and the initial conditions are like $n_{1}=10^{3},n_{k\neq 1}=0,N_{p}^{H^{+}}=10^{5},N_{p}^{L}=2.5\times 10^{4},N_{c}^{L}=7.5\times 10^{4},N_{c}^{H^{+}}=10^{3}$.

The above is the case of *ξ* = 0, while the following Fig 8(a) and 8(b) are the case of *ξ* = 1, which are surprisingly not similar, unlike the similarity of the previous Fig 7(a) and 7(b).

A closer observation reveals that although the equilibrium time images of lactose are disparate, the equilibrium time images of H^+^ are still similar in shape. This is primarily due to the fact that in the presence of leakage, as mentioned earlier, the equilibrium of the two kinds of particles has little influence on each other. And no matter initial concentration or initial particle numbers is fixed, in the range of periplasm volume fraction in our calculation (*V*_*p*_/(*V*_*p*_ + *V*_*c*_) ∈ [0.1, 0.9]), the net transport direction of hydrogen ions is from periplasm to cytoplasm according to Δ*G*. Increasing the periplasm volume proportion in the presence of leakage, when the initial concentration is fixed, the (initial) net transport rate remains constant and the net transport amount increases, while when the initial particle number is fixed, the (initial) net transport rate decreases and the net transport volume does not change much from the aforementioned proportionality of the equilibrium solution, so the time required to reach equilibrium in both cases shows a monotonically increasing state.

The case of lactose is more complicated, especially in the condition of fixed initial particle number, where there are three inflection points and also the order for H^+^ and lactose to reach equilibrium changes with different volume fraction of periplasm. We will give the explanation for the minimal value point generated near *V*_*p*_/(*V*_*p*_ + *V*_*c*_) = 0.25 under the condition of fixed initial particle numbers later in the discussion section. Regarding the remaining phenomena embodied in the two figures, it is difficult for the author to give a reasonable explanation here, and I can only leave it to the experimental data to verify or deny.

### A calculation example of an open system

All the previous calculations are based on closed systems, meaning that the substrate concentration in periplasm changes as in cytoplasm. But in fact both the original model and our extended model can calculate the open system case, and such a constant substrate concentration in periplasm is sometimes closer to the reality. To adapt the model for the open system, we only need to remove the two equations about $dN_{p}^{A}/dt,dN_{p}^{B}/dt$ from the 10 master equations and make $N_{p}^{A},N_{p}^{B}$ always equal to the initial value. And in the actual calculation, it is only necessary to discretize the time as we did before and let $dN_{p}^{A}/dt,dN_{p}^{B}/dt$ take the initial value again after running the original model for one time step. In the following, we try to reproduce the results of an experiment in article [19] using the open system extended model.

**Fig 9:**
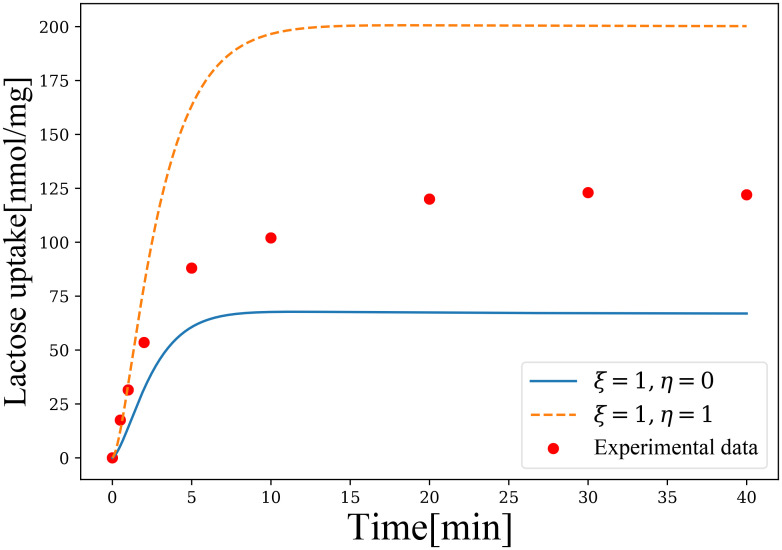
Lactose translocated into cytoplasm by *E*. *coli* T184 wild-type lactose permease. The horizontal axis of the graph indicates the time *E*. *coli* T184 cells were incubated under specific conditions, and the vertical axis shows the increase in lactose concentration in cytoplasm compared to the initial condition. The red dots are the experimental data extracted from the article of Ujwal *et al*[19]. The solid and dashed lines are the results of our simulations when *ξ* = 1, *η* = 0 and *ξ* = 1, *η* = 1, respectively.

The value of the parameters set for simulations are re-estimated by referring to the relevant results in [13, 20]. Although both simulation curves in Fig 9 are far from the experimental data, we have previously found a linear relationship between the steady-state solution and *η*, so it can be expected that the existence of a *η* ∈ [0.4, 0.5] will bring the fitted curve much closer to the experimental data. In fact, not only *η*, but we can also adjust *ξ* and thus change the time required to reach equilibrium appropriately to make the simulation curve more closely match the experimental data, and the specific calculation will be very mathematical thus omitted here.

## Discussions of the above results

As we said in the last section, we first give the results of all simulations together, and then analyze the data and discuss the results. In the following, we will analyze the results separately for each subsection above.

### Effect of parameters *ξ* and *η* (leakage intensity) on equilibrium properties

It is obvious from the above Fig 2 that *ξ* always triggers a dramatic change in the quantities we observe as it changes from zero to positive, while *η* only causes smooth changes no matter *η* = 0 or not. What’s more, when *ξ* → 0^+^, that is to say when *ξ* is down to 0 from 1, the time required for the system to stablize *T* → ∞. After excluding the *ξ* = 0 cases, it can also be found that the effects of *ξ* and *η* on the equilibrium solution ($N_{p}^{H^{+}}/N_{c}^{H^{+}}$ and $N_{p}^{L}$/$N_{c}^{L}$) are always opposite, one positively correlated and the other negatively correlated. In contrast, the impacts of *ξ* and *η* on the equilibrium time for H^+^ and lactose are more complex. The former is easy to explain, because *ξ*, *η* are both monotonically and almost linearly related to $N_{p}^{H^{+}}/N_{c}^{H^{+}}$ and $N_{p}^{L}$/$N_{c}^{L}$, but as we show later, $N_{p}^{H^{+}}/N_{c}^{H^{+}}$ and $N_{p}^{L}$/$N_{c}^{L}$ remain almost constant when *ξ* = *η*, so one of *ξ*, *η* must have a positive effect and the other negative.

From the above Fig 3(a) and 3(b), it can be found that when *ξ* is not very small (≥ 0.1), the above general conclusions about the equilibrium solution (insignificant variation of the equilibrium solution with *ξ* and definite proportionality between the equilibrium solutions) are correct within error permissibility. In fact, when the total number of cotransporters is increased (in this example we just need to increase *n*_1_), the error will be significantly reduced and the proportional relationship between the equilibrium solutions will become more obvious and precise.

Now in the special case of *ξ* = *η* ≠ 0, we can we analyze the variation of the equilibrium time, which can be inferred by splitting the full reaction cycle into two independent transport process. As can be seen in Fig 3(c), the time for H^+^ to reach equilibrium is always shorter than the time for lactose when *ξ* ≠ 0. Because we can formally split the overall transort process in the presence of leakage, the full reaction process can be viewed as a combination of a leakage-free cotransport process and a separate uniport process of H^+^, with little interference between them. Then H^+^ has two transport processes transporting it, while lactose has only one. So H^+^ can reach equilibrium with the net transport of the two processes canceling each other, while lactose has not reached equilibrium at that point. It can also be observed that the time for the two kinds of particles to reach equilibrium do not have a consistent trend with the change of *ξ*. Both of them are large when *ξ* is small, after that it decreases rapidly with the increase of *ξ*, and the change is not obvious after *ξ* > 0.2. But the equilibrium time of lactose has a small rebound after *ξ* > 0.5, while H^+^ continues to remain monotonically decreasing. The reason for this phenomenon is not clear, and it is speculated that there may be some calculation errors. Another point worth mentioning is that particle numbers converge much faster when *ξ* = 0 than *ξ* ≠ 0. The addition of the transition between cotransporter states 2 and 5 (*i*.*e*., making *ξ* ≠ 0) makes the convergence of the system much slower.

Finally we will return to the discussion of the difference between the two parameters *ξ* and *η*. In the above example, we have illustrated the different effects of the two parameters *ξ* and *η* on some equilibrium properties. Although the different effects of *ξ* and *η* appear to be related to the gap between the initial state and the equilibrium state, that is, the different effects of *ξ* and *η* on the equilibrium solution and time may change when the initial condition changes (the equilibrium solution changes accordingly). For example, one might think that the reason for the abrupt change caused by parameter *ξ* but not *η* near zero is that the net transport of H^+^ is from periplasm to cytoplasm and increases dramatically as parameter *ξ* and *η* begin to change from 0, so that *ξ*, which controls the cotransporter state 2 to 5 transition, might respond more significantly to this. However, after we actually change the initial conditions to produce a change in the net transport direction of H^+^, we still find that only *ξ* causes a sudden change near 0. This phenomenon may imply that the different effects of *ξ* and *η* may be determined by more intrinsic factors such as *α*_*ij*_.

### Effect of periplasm and cytoplasm volumes *V*_*p*_, *V*_*c*_ on equilibrium properties

The above phenomena shown in Fig 4 can be explained from the theoretical analysis, for which we consider the Gibbs free energy variation at the starting moment Δ*G*. For this cotransport process of the *E*. *coli* LacY protein, we can rewrite the free energy variation in terms of the sum of the chemical potential multiplying the stoichiometry of the two particles [21], bringing the data and slightly transforming then we have,

$$\begin{array}{c} \Delta G=k_{B}T\;\text{ln}(\frac{N_{c}^{H^{+}}N_{c}^{L}}{N_{p}^{H^{+}}N_{p}^{L}})+Q_{H^{+}}\Delta \Psi +2k_{B}T\;\text{ln}\left(V_{p}/V_{c}\right). \end{array}$$

For a fixed initial condition, the first of the three terms on the right-hand side of the above equation is constant, and based on the data we use in calculation and the data corresponding to the environment in which *E*. *coli* is usually found, this term is usually in the (−4*k*_*B*_ *T*, *k*_*B*_ *T*) interval, and the second term is usually around −3.9*k*_*B*_ *T* [22]. When changing *V*_*p*_ or *V*_*c*_, the absolute value of Δ*G* can be reduced only if *V*_*p*_/*V*_*c*_ > 1, and making |Δ*G*| decrease produces a larger rate of change than making it increase when the amount of change in *V*_*p*_/*V*_*c*_ is the same. The equilibrium solution is reached when Δ*G* is 0, and it is easy to find that Δ*G* varies monotonically during the cotransport process. So Δ*G* can actually represent the “gap” between the initial state and the equilibrium state, and the gap between the equilibrium solutions is larger when the difference between Δ*G* is larger. And since the negative membrane potential in this process contributes positively (also not small proportion) to Δ*G*, it produces a very different property when the membrane potential decreases or even reverses. However, as is well known, in actual *E*. *coli* cells *V*_*p*_/*V*_*c*_ < 1, so it is known from the above analysis that changing *V*_*c*_ or *V*_*p*_ would have little effect on the equilibrium solution and net transport.

Also, the phenomenon shown in Fig 5 can still be explained using the free energy variation Δ*G*, because although Δ*G* is constant as particle concentration is constant, the net change of particles transported through the cotransporter is different as the number of particles involved in cotransport expands with *V*_*p*_ or *V*_*c*_ increasing. Based on the initial conditions we give here, the H^+^ concentration is much higher in periplasm than in cytoplasm, and lactose concentration is a bit higher in cytoplasm than in periplasm. In the case of *ξ* = 0, the net transport is a 1:1 cotransport for both kinds of particles from periplasm to cytoplasm, so when periplasm volume *V*_*p*_ fixed and cytoplasm volume *V*_*c*_ varied, the net transport does not change much and therefore the time to reach equilibrium does not change significantly. But in contrast the net transport increases almost linearly when *V*_*c*_ fixed and *V*_*p*_ varied, so that the time required to reach equilibrium also increases monotonically with the volume ratio *V*_*p*_/*V*_*c*_.

The two phenomena in Fig 6 are quite anomalous, but they can still be broadly explained as follows. Since the concentration of H^+^ and lactose on both sides of the cell membrane and the number of cotransporters are all constant, the transport rate of the cotransporter changes little when varying volume ratio, so the main difference in the equilibrium time comes from the number of particles by net cotransport. The H^+^ concentration is considerably higher in periplasm than in cytoplasm, with lactose concentration the opposite. Therefore, according to the proportional relationship that needs to be satisfied by the equilibrium solution, the net transport of H^+^ is from periplasm to cytoplasm, which eventually makes the H^+^ concentration in cytoplasm much higher than in periplasm. Then increasing *V*_*p*_ causes the net transport of H^+^ to increase significantly, so the equilibrium time also increases significantly and monotonically with *V*_*p*_/*V*_*c*_ up. Meanwhile, the net transport of lactose is from cytoplasm to periplasm, so the effect of changing *V*_*p*_ on the net transport of lactose is positive, and therefore the equilibrium time increases with *V*_*p*_/*V*_*c*_ up, but asymptotic phenomena occur as *V*_*p*_/*V*_*c*_ continues to increase. Thus, the increasing rate of the time for lactose to reach equilibrium slows down rapidly as *V*_*p*_/*V*_*c*_ increases, but is still greater than the H^+^ equilibrium time.

When varying *V*_*c*_, the case of H^+^ is similar to the earlier discussion and easily explained as monotonically decreasing with *V*_*c*_/*V*_*p*_, but the case of lactose is more complicated. Due to the proportionality of the equilibrium solution, when *V*_*c*_ is very large or small, the concentration of lactose in cytoplasm at equilibrium will be close to the initial concentration in cytoplasm or periplasm, respectively, so that the net transport is similar and both are small, but *V*_*c*_/*V*_*p*_ of moderate size may produce a large net transport. This may lead to a phenomenon that equilibrium time of lactose increases and then decreases with *V*_*c*_/*V*_*p*_ up as shown in Fig 6(b), but the exact situation still needs to be supported by experimental data. From the above discussion we can find that in the case of *ξ* ≠ 0, the correlation between the equilibrium of the two particles is very weak, not only the equilibrium solution itself is irrelevant, but also the correlation of the time required to reach the equilibrium solution is very low, which is exactly an important property of this model. This leads a slightly questioning of the model when *ξ* ≠ 0.

Now it comes to Fig 7. With a fixed initial concentration, the H^+^ concentration in the cytoplasm increases continuously as the volume fraction of periplasm increases due to the higher concentration of H^+^ in periplasm than in cytoplasm in the initial conditions. However, the increase does not exceed the initial H^+^ concentration in periplasm, meanwhile the volume of cyptoplasm decreases accordingly. Therefore, the time to reach equilibrium is likely to increase when the periplasm fration remains in 8%-40% at constant initial concentration and constant cotransport rate.

With a fixed initial particle number, |Δ*G*| decreases as the fraction of periplasm increases, resulting in a decrease in the net amount of particles transported, but the change in volume also decreases the transport rate of cotransporters from periplasm to cytoplasm but increases the rate of reverse direction, with a decrease in the (initial) net transport rate. A qualitative analysis of the form reveals a more pronounced change in the net transport rate (than in the net amount of particles transported), but the change rate of the net transport rate gradually decreases as the fraction of periplasm increases, thus possibly leading to an increase in the time required to reach equilibrium when the periplasm fration in 8%-40%. The above analyses and explanations are qualitative and still need to be verified or denied by experimental data.

We will finally explain the appearance of the minimal value point in Fig 8. Since the initial value of $N_{c}^{L}/N_{p}^{L}=1/3$, when *V*_*c*_/*V*_*p*_ is around 3, the initial condition of lactose is actually very close to the equilibrium state because the total number of cotransporters is small compared to the number of particles. $N_{c}^{L}$ and $N_{p}^{L}$ change very little during the cotransport process and can be considered as reaching equilibrium very early. Changing the initial value of lactose and computationally verifying, it is found that this phenomenon is practically universal. When the volume ratio is adjusted so that the initial value of lactose is close to the equilibrium solution, there is always a significant reduction in the time for lactose to reach equilibrium, causing lactose’s reaching equilibrium before H^+^. More generally, a similar phenomenon occurs for both kinds of particles. Nevertheless, it should still be noted that for the general realistic initial values, as in the previous analysis, H^+^ usually reaches equilibrium before lactose because of the additional leakage current regulation, which also complements and explains the above inference.

### A calculation example of an open system

Here we will explain the difference between the original model and the extended model in the problem of simulating the above experimental data. As we discussed earlier, for a fixed initial condition, the original system has only two different equilibrium solutions for *ξ* = 0 and *ξ* ≠ 0, and adjusting *ξ* when *ξ* ≠ 0 can only change the rate of convergence to the steady-state solution. Such a feature is very detrimental to fitting realistic experimental data, for it is easy to have the problem that the simulation is too far from the experimental data but there is no way to adjust them. For the extended model, both the equilibrium solution and the time required to converge to the equilibrium solution can be adjusted by *ξ* and *η*, which does have some advantages in reproducing the experimental data. However, finally, we have to point out that a careful observation reveals that the simulation curve produces a slight downward convexity at the beginning of the symport, which is not found in the experimental data. So even with the extended model, the experimental data cannot be perfectly reproduced, and the model still has much room for improvement.

## Conclusions and further discussions of the above results

We start the article with a certain extension of the model in the original article [14], then show that the extended model still satisfies the static head equilibrium condition, and therefore such an extension is appropriate. Then we compare the difference between our new parameter *η* and the original parameter *ξ* of similar status in the model in detail, and then show that our changes are valuable. We find that *η* linearly affects the time required to reach equilibrium for the two particles differently, but the effect of *ξ* is discontinuous at the zero point, and even when the zero point is removed, *ξ* is only negatively but not linearly correlated with the equilibrium time. However, for the equilibrium solutions of $N_{p}^{H^{+}}/N_{c}^{H^{+}}$ and $N_{p}^{L}$/$N_{c}^{L}$, excluding the abrupt change caused by *ξ* at the zero point, both *η* and *ξ* produce a linear effect and their effects are always opposite.

After that, we have studied and verified a specific computational example. For the cotransport process of *E*. *coli* LacY protein symporting H^+^ and lactose, we apply the random-walk model proposed in article [14] (which is the special case of *ξ* = *η* in our extended model) to computationally simulate and find, validate or predict the following phenomena.

- In the absence of leakage, the concentrations of the two particles H^+^ and lactose reach stability simultaneously; in the presence of leakage, the time required for the two particles to reach the equilibrium state is separated. In the realistic situation where the number of cotranporters is small relative to the number of transported particles, the intensity of leakage, if leakage exists, hardly affects the equilibrium state of cotransport, but affects the time for both particles to reach the equilibrium state, and generally the more pronounced leakage is, the shorter the time to reach equilibrium.
- In the absence of leakage, fixing the initial number or concentration of H^+^ and lactose in periplasm and cytoplasm, for *E*. *coli* cells in the same strain with different periplasm and cytoplasm volumes, the volume of periplasm and cytoplasm has little effect on the equilibrium solution, and the periplasm volume but not the cytoplasm volume has a greater effect on the time required to reach equilibrium. In other words, when periplasm volumes are similar and cytoplasm volumes are different, cells have similar equilibrium states and need similar time to reach equilibrium by this cotransport process, but not vice versa.
- In the presence of leakage, fixing the initial concentration of H^+^ and lactose in periplasm and cytoplasm, for *E*. *coli* cells in the same strain with different periplasm and cytoplasm volumes, the time required for the pH of cytoplasm to stabilize increases monotonically with the periplasm to cytoplasm volume ratio increasing. Meanwhile, the time for the lactose concentration to reach equilibrium has a more complex relationship with the volume ratio of periplasm and cytoplasm as shown in Fig 6.
- For a certain *E*. *coli* cell (or cells with similar size from the same strain), fixing the initial number or concentration of H^+^ and lactose in periplasm and cytoplasm, the time for the pH of cytoplasm to stabilize increases monotonically as the cell loses water regardless of the presence or absence of leakage (under the condition of cell survival), whereas the time for the concentration of lactose to stabilize varies more complexly, as seen in Figs 7 and 8.

Many of the above phenomena can be qualitatively explained, but some of them still can not be well explained and need to be verified or negated by experimental data. Then we adapt the extended model for a open system and reproduce the experimental data from the literature [19] to some extent.

After summarizing the results above, it is easy to notice that in the case of the parameter *ξ* = *η* = 0, *i*.*e*., no leakage, the results are mostly consistent with biological or chemical intuition, but the appearance of some properties for *ξ* = *η* ≠ 0 cases is a little anomalous. The biggest problem is the equilibrium solution. When *ξ* = *η* ≠ 0, the equilibrium solution almost strictly satisfies the concentration proportionality under the general condition that the number of cotransporters is much smaller than the number of particles to be transported. For the symport of *E*. *coli* LacY protein, the concentration of lactose in periplasm and cytoplasm at equilibrium are almost equal because the lactose molecule is neutral, and this relationship does not change with other conditions such as the initial value. In contrast, hydrogen ions will accumulate in large quantities into the *E*. *coli* cells. These phenomena may be somewhat different from the reality. Actually for this model in *ξ* = *η* ≠ 0 cases, *i*.*e*. the original model, any neutral particle involved in cotransport has equal concentration in periplasm and cytoplasm, which is no different from ordinary diffusion and far from the energy-consuming active transport.

Next, from a kinetic point of view, there are still some parts of the model that need discussions when the parameter *ξ* ≠ 0. Although the essence of the model is cotransport, we find that the correlation of the time required for two particles to reach equilibrium is not strong. If the initial state of a certain particle happens to satisfy the proportionality relation that the equilibrium state should satisfy, it will soon equilibrate and is hardly controlled by the other particle. From this perspective, the two kinds of particles are almost independent of each other. Here, the effect of H^+^ on lactose transport is more like a facilitated transport, which is also used to explain the uncoupled cotransport in recent years, such as literature [6, 23]. Facilitated transport may seem to provide a reasonable explanation for the above “anomalies”, but it actually does not consider one of the special cases. Calculations using the original model show that when *ξ* ≠ 0 and the concentrations of H^+^ and lactose molecules in periplasm and cytoplasm are close, the concentration of lactose molecules will not change much during symporting, but H^+^ will spontaneously accumulate in cytoplasm. The large gap between such results and reality indicates that there are still some problems with the original model.

So can our extended model solve the above problem? As shown in Fig 2, we already know that the new parameter *η* acts as a linear perturbation on the basis of the equilibrium state resulting from parameter *ξ*. However, it is clear that the effect of *η* on the equilibrium solution is not obvious in the *ξ* = 0 case, but only the *ξ* = 0 cases are consistent with our expectation that H^+^ do not actively aggregate intracellularly, while *ξ* ≠ 0 almost certainly leads to a large influx of H^+^ into cytoplasm. Therefore, the extended model does not provide a big improvement with respect to the problems mentioned above.

We now turn to the problem concerning the estimation of *ξ* and *η*, and what we consider to be an important shortcoming of the extended model. In the original model, the parameter *ξ* has a certain biological significance and represents the strength of the leakage current, so the value of *ξ* can be estimated by experimentally determining the leakage current and maximal cotransport current. However, when the model is extended, the biological significance of *ξ* and *η* is greatly reduced, and it is difficult to tell the relationship between them and the leakage current, so the above estimation method basically fails. Therefore, we believe that for the extended model, we can only use it to reproduce the experimental data under various conditions and select the value of the best-fitting parameter *ξ*, *η* as the reference value. But mathematically speaking, this may produce the problem of different reference values under different experimental conditions or even large gaps, which is still an unsolvable problem and a direction for further improvement of the model.

Furthermore, actually there is no experimental evidence showing this uncoupled sugar translocation (“leakage”) could happen before the concentrations of lactose across the cytoplasmic membrane reach equilibrium. In other words, all the above calculations and explanations are based on a shared assumption that the symport and uncoupled sugar translocations are simultaneous and interact with each other. Such an assumption treats the observed symport in the absence of H^+^ electrochemical gradient as a leakage as well, but if one considers that the driving forces of symport are different in the presence and absence of the electrochemical gradient, and thus they are two different kinds of transport, then the assumption of a simultaneous leakage with symport is not accepted, which still makes sense. Namely, when there is an electrochemical gradient of H^+^ across the membrane, it drives a perfect coupled symport of lactose and H^+^, then the cotransporters continue to work to transport lactose molecules when the pH is equal on both sides of membrane, reaching a new equilibrium state. Therefore, as we did before, there is still no way to discard the case of *ξ* = *η* = 0. It may be possible in the future to add subsequent leakage processes to the case *ξ* = *η* = 0 to form a new model, which could provide a better explanation of uncoupled sugar translocation while retaining the advantage of a being more consistent with biological and chemical intuition, but this would be beyond the scope of this paper to explore.

Therefore, although changing the parameter *ξ* gives the model good kinetic properties, such as adjusting *ξ* to control the speed of convergence, and the new parameter *η* partly solve the problem of the gap between the original model results and reality, the model still has some defects. The parameters *ξ*, *η* is introduced to solve the problem of leakage current, but this solution is not perfect, and we may still need to think about other solutions. Maybe the specific and accurate mathematical description of the symport of cotransporter LacY still lacks knowledge.

## Supporting information

S1 File (PY)

S1 Graphical abstract (PDF)
