# Supplementary figures and images for: Equilibrium properties of E. coli lactose permease symport—A random-walk model approach
Source: PLoS One. 2022 Feb 4;17(2):e0263286. doi: 10.1371/journal.pone.0263286 (PMC8815909; doi:10.1371/journal.pone.0263286)

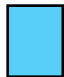

Lactose permease (LacY protein)

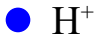

$H^+$

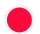

Lactose

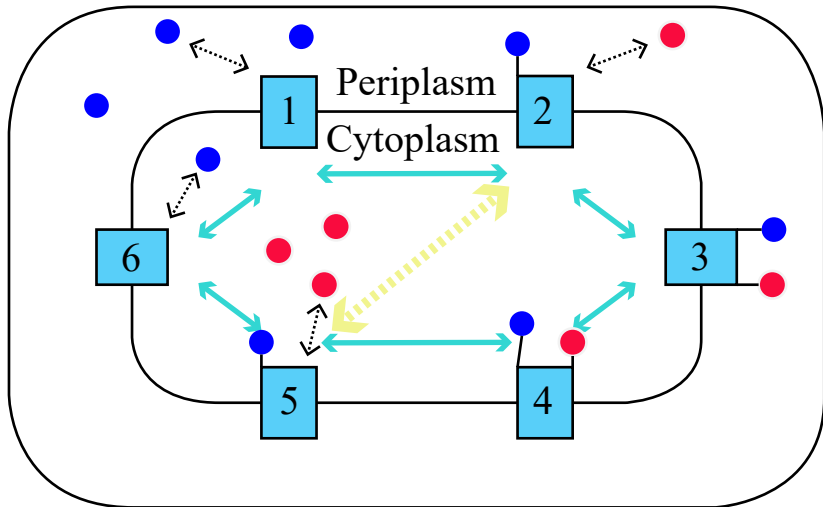

Supplement: S1 Graphical abstract — (PDF) [file pone.0263286.s002.pdf]
